# Koumine Attenuates Lipopolysaccaride-Stimulated Inflammation in RAW264.7 Macrophages, Coincidentally Associated with Inhibition of NF-κB, ERK and p38 Pathways

**DOI:** 10.3390/ijms17030430

**Published:** 2016-03-22

**Authors:** Zhihang Yuan, Froilan Bernard Matias, Jing Wu, Zengenni Liang, Zhiliang Sun

**Affiliations:** 1 Department of Clinical Veterinary Medicine, College of Veterinary Medicine, Hunan Agricultural University, Changsha 410128, China; yuanzhihang84@163.com (Z.Y.); wu23jing@aliyun.com (J.W.); 2 Hunan Co-Innovation Center for Utilization of Botanical Functional Ingredients, Changsha 410128, China; 3 Department of Animal Management, College of Veterinary Science and Medicine, Central Luzon State University, Science City of Muñoz, Nueva Ecija 3120, Philippines; loydyreyesmatias@gmail.com; 4 Department of Hunan Agricultural Product Processing Institute, Changsha 410128, China; enni_007@163.com

**Keywords:** koumine, inflammation, LPS, NO, iNOS, NF-κB, RAW264.7 macrophages

## Abstract

Medicinal herbal plants have been commonly used for intervention of different diseases and health enhancement worldwide. Koumine, an alkaloid monomer found abundantly in *Gelsemium* plants, can be effectively used as an anti-inflammatory medication. In this study, the mechanisms associated with the preventative effect of koumine on lipopolysaccharide (LPS)-mediated inflammation in RAW264.7 macrophages were investigated. Koumine induced a decrease in the level of inducible nitric oxide synthase (iNOS) protein, concomitant reduction in the production of nitric oxide (NO) and reduction of the levels of interleukin (IL)-6, tumor necrosis factor-α (TNF-α) and IL-1β. Furthermore, koumine decreased the phosphorylation of p65 and inhibited nuclear factor κ Bα (IκBα) proteins, resulting in lower production of nuclear factor (NF)-κB transactivation. Koumine also induced a decrease in the phosphorylation of extracellular-signal-regulated kinases (ERK) and p38 in RAW264 cells. In conclusion, these findings reveal that koumine decreases the productions of pro-inflammatory mediators though the suppression of p38 and ERK MAPK phosphorylation and the inhibition of NF-κB activation in RAW264.7 cells.

## 1. Introduction

Macrophages play a crucial role in innate immunity and inflammatory responses. Various stimuli can stimulate macrophages to produce chemokines and pro-inflammatory cytokines, particularly macrophage inflammatory protein-2 (MIP2), IL-6, NO, IL-1β, tumor necrosis factor-α (TNF-α), IL-10, and inducible nitric oxide synthase (iNOS) [1].

Nitric oxide (NO) is a free radical synthesized from L-arginine by three NO synthases (NOSs), iNOS, neuronal NOS (nNOS) and endothelial NOS (eNOS), and is involved in different physiological functions of the body such as regulating inflammation, mitochondrial function, cell death, and neurotransmission [2,3]. iNOS has been shown to be the major NO synthase involved in NO production during inflammatory conditions [4]. However, excessive production of NO at the site of inflammation can trigger most cell functions such as signal transduction, mitochondrial functions, excessive pro-inflammatory mediator production and cell apoptosis, which can further increase local tissue damage [5]. Studies showed an obvious pertinence between NO production and some serious inflammatory diseases including systemic lupus erythematosus (SLE) [6] and rheumatoid arthritis (RA) [7]. The differences observed in the expression of iNOS and formation of NO among RA patients [5] can be associated with the highly (CCTTT)*n* pentanucleotide repeats at the *iNOS* gene promoter region, which is involved in regulation of iNOS transcription [8]. In addition, susceptibility to SLE has been presented by some studies [9,10] to be associated with polymorphism of the *iNOS* gene. Thus, NO and iNOS are crucially involved in the inflammatory process.

Previous studies consider that nuclear factor (NF)*-*κB activation has been proposed to play an important role in iNOS induction [11]. The NF-κB family contains five structurally related members consisting of RelA (p65), RelB, c-Rel, p50 and p52. Normally, these structurally related proteins dimerized (homo or hetero) forming transcriptionally repressive (e.g., p50-p50) or active (e.g., p50-p65) NF-κB dimers, inactively sequestered in the cytoplasm by the inhibitory κB (IκB) family of proteins, which include IκBα, IκBβ, p105 and p100 [12]. Following the separation of receptor families such as LTβR, TNFR, TCR, BCR, IL-1R, and RANK, the molecular component and signaling events induced by NF-κB activation pathways activate many intermediate kinases (e.g., IRAK, NIK, and RIP) and recruit scaffolding and adaptor proteins (e.g., TRAFs, TIRAP, and MyD88). Various stimuli such as LPS, TNF-α, double-stranded (ds) RNA and ultra-violet radiation can activate classic NF-κB, which consists of a p65 (RelA) and p50 heterodimer. These factors bind to receptors on cell membrane and trigger complex signal transduction pathways that cause activation of the IκB kinase (IKK) [13]. Activated IKK, in return, accelerates phosphorylation of IκB, which are further ubiquitinated and degraded through proteolysis by proteasome. Degraded IκB results in the release and translocation of p-p65 into the nucleus where it binds to the promoter region of inflammatory genes for transcriptional regulation [14,15,16].

*Gelsemium* is a small genus of the family Loganiaceae that is comprised of three commonly known species: *Gelsemium sempervirens* (*G. sempervirens)* Ait. And *Gelsemium rankinii* (*G. rankinii*) Small, which are both indigenous to North America, and *Gelsemium elegans* (*G. elegans*) Benth., which is indigenous to China and Southeast Asia. *G. sempervirens* has been used in traditional medicine to reduce pain and anxiety and has been reported to reduce anxiety *in vivo*, even in very low dose. On the other hand, even though *G. elegans* can be toxic to humans and animals, it has been used in Chinese medicine to reduce or counter pain, inflammation and neoplasms [17,18]. Koumine is a kind of alkaloid that forms the major active components of *G. elegans.* Studies showed that crude alkaloid extracts, administered intraperitoneally, has notable potential as an anti-tumor [19] and analgesic [20] agent. However, koumine’s anti-inflammatory effect and mechanism is not clearly understood.

In this study, koumine’s anti-inflammatory effects in LPS-stimulated RAW264.7 were examined and its potential anti-inflammatory mechanisms were investigated. The results revealed that koumine influences LPS-mediated pro-inflammatory cytokines by interfering with NF-κB, p38 and ERK signaling pathways.

## 2. Results

### 2.1. Koumine Suppresses the Levels of NO and iNOS in LPS-Stimulated RAW264.7 Cells

**Figure 1:**
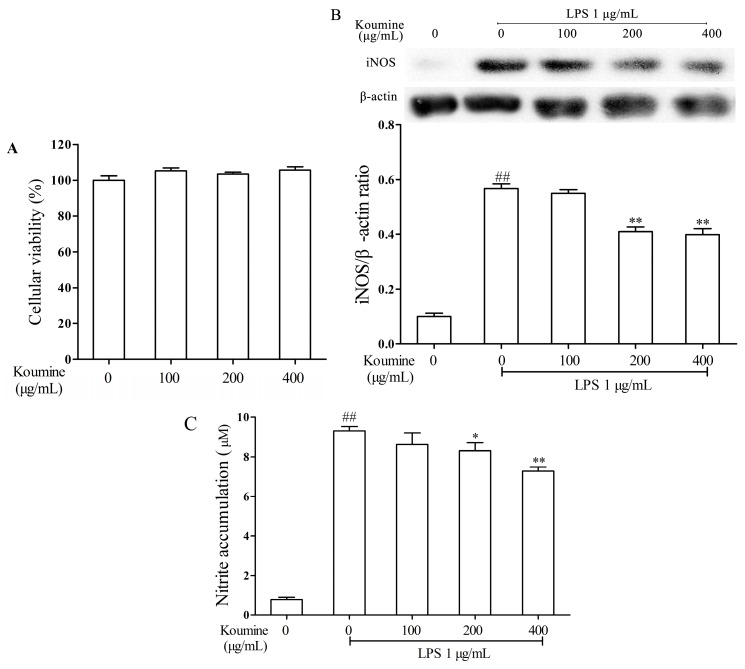
Effects of koumine on lipopolysaccharide (LPS)-induced inducible nitric oxide synthase (iNOS) and nitric oxide (NO) levels in RAW264.7 cells. (**A**) RAW264.7 macrophages (5 × 10^3^ cells/well) were pre-incubated with 100, 200 or 400 μg/mL koumine for 24 h. Cytotoxicity of koumine on RAW264.7 (*n* = 5) was evaluated using MTT assay; (**B**) RAW264.7 macrophages (5 × 10^5^ cells/well) were pre-exposed to 100, 200 or 400 μg/mL koumine for 1 h. Subsequently, cells were treated with 1 μg/mL LPS in pre-incubated mediums. After incubation for an additional 18 h, the protein levels of iNOS were determined by Western blotting; the quantification histogram of iNOS protein expression was normalized by β-actin. (*n* = 3); and (**C**) Culture media were isolated and the culture supernatants were collected for NO detection by Griess reagent. The data were expressed as mean ± standard deviations (SD) (*n* = 3). ^##^ *p <* 0.01 *vs.* control group; * *p <* 0.05 and ** *p <* 0.01 *vs.* LPS group.

MTT (3-(4,5-dimethylthiazol-2-yl)-2,5-diphenyltetrazolium bromide) assay was performed to determine the inhibitory effect of koumine on RAW264.7 macrophage cells. As shown in Figure 1A, after 24 h incubation with different concentration of koumine (0, 100, 200 and 400 μg/mL), no significant effects on cell viability were observed, suggesting that koumine has no inhibitory effect on the growth of RAW264.7 macrophages. Thus, all concentrations were selected for further investigation.

Koumine significantly inhibited production of NO and expression of iNOS in LPS-treated RAW264.7 macrophages. The inhibitory effects of koumine were noticeable at 200 μg/mL concentration, while its maximum effect was observed at 400 μg/mL concentration. With no LPS treatment, koumine alone produced no significant influence on NO production and iNOS expression in RAW264.7 macrophages.

As shown in Figure 1B, LPS evidently increased the expression of iNOS. Koumine significantly decrease the expression level of iNOS in LPS-stimulated RAW264.7 macrophages where the maximum decreases were obtained at the two high concentrations of koumine (200 and 400 μg/mL). Reduction of NO production was shown to be directly related to the concentration of koumine compared to LPS-stimulated RAW264.7 macrophages. Particularly, the maximum NO reduction was obtained at the highest koumine concentration (400 μg/mL) (Figure 1C).

### 2.2. Koumine Attenuates the Production of Pro-Inflammatory Cytokines in LPS-Treated RAW264.7 Macrophages

**Figure 2:**
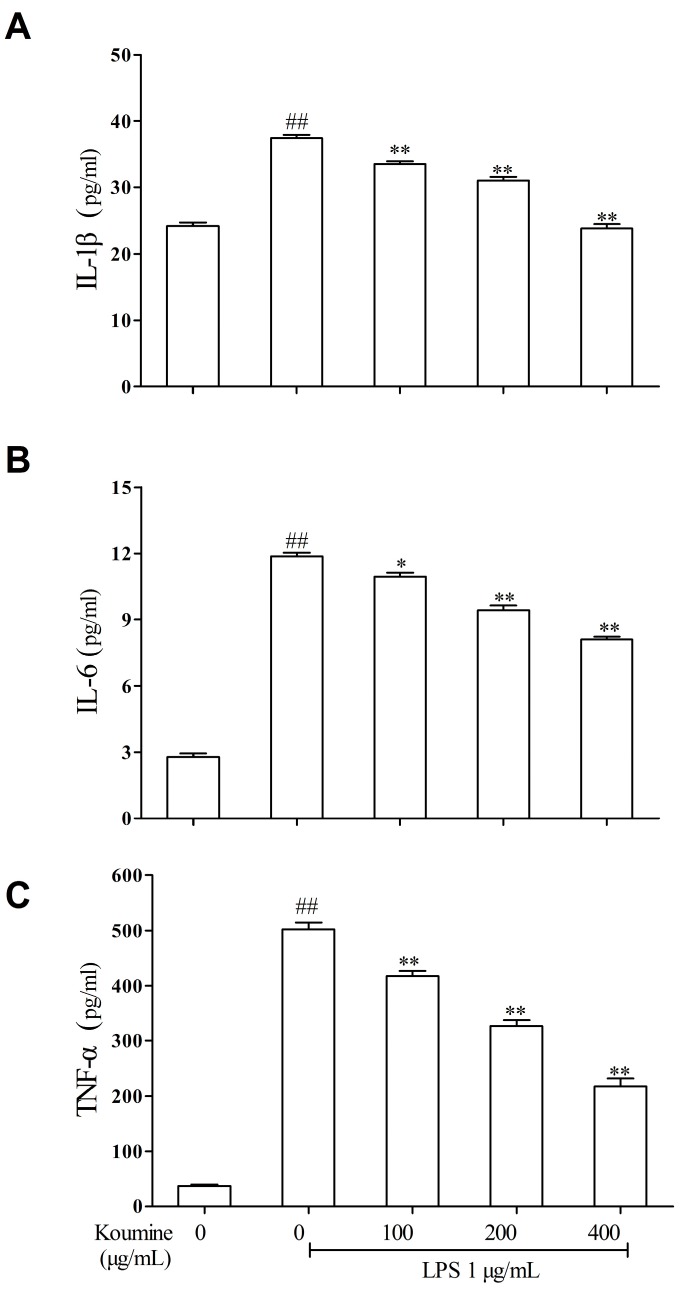
Inhibitory effect of koumine on the pro-inflammatory cytokine production in RAW264.7 cells. The production of: (**A**) interleukin (IL)-1β; (**B**) IL-6; and (**C**) tumor necrosis factor-α (TNF-α) were assayed in the culture medium of cells stimulated with 1 μg/mL lipopolysaccharide (LPS) for 18 h in the presence of 100, 200 or 400 μg/mL koumine. After culture supernatants were collected, equal volume of cell-free culture media (100 μL) was reacted with Griess reagent and the absorbance at 540 nm was measured. The data were expressed as mean ± standard deviations (SD) (*n* = 3). ^##^ *p <* 0.01 *vs.* control group; * *p <* 0.05 and ** *p <* 0.01 *vs.* LPS group, respectively.

The extent of inflammation and recruitment of immune cells associated with the development of inflammatory processes are influenced by inflammatory cytokines [18]. The productions of different inflammatory cytokines were determined using enzyme-linked immunosorbent assay (ELISA) kits. As shown in Figure 2, overproduction of IL-1β, IL-6 and TNF-α in RAW264.7 macrophages were generally observed after stimulation with LPS for 24 h. On the other hand, koumine hampered IL-1β, IL-6 and TNF-α production in LPS-stimulated RAW264.7 macrophages in a concentration-dependent manner.

### 2.3. Koumine Inhibits p38 and ERK Phosphorylation in LPS-Stimulated RAW264.7 Macrophages

**Figure 3:**
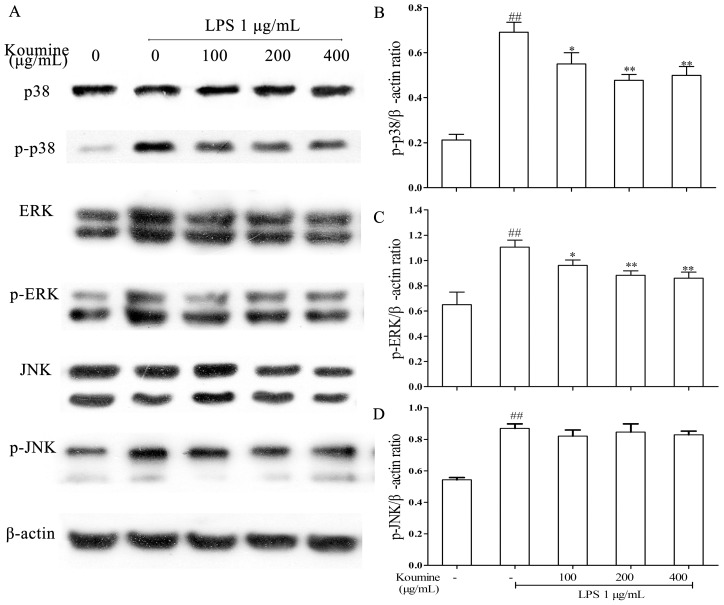
Effect of koumine on the expression of MAPK signaling pathway in LPS-stimulated RAW264.7 cells. Cells were pretreated with 100, 200 or 400 μg/mL koumine for 1 h, and treated with 1 μg/mL LPS in the mediums containing koumine for 18 h. Equal amounts of protein in cell lysates were analyzed by Western blot. The β-actin protein levels were used to confirm that equal amounts of protein were subjected to electrophoresis. (**A**) The expression levels of phosphorylation p38 (p-p38), phosphorylation extracellular signal-regulated protein kinase (p-ERK), and phosphorylation c-Jun terminal kinase (p-JNK) proteins; (**B**) The quantification histogram of p-p38 protein expression normalized by β-actin; (**C**) The quantification histogram of p-ERK protein expression normalized by β-actin; and (**D**) The quantification histogram of p-JNK protein expression normalized by β-actin. The data were expressed as mean ± standard deviations (SD), (*n* = 3). ^##^ *p <* 0.01 *vs.* control group; * *p <* 0.05 and ** *p <* 0.01 *vs.* LPS group, respectively.

MAPKs play a critical role in the regulation of cell growth, differentiation, and control cellular responses to cytokines. The phosphorylation of ERK, JNK, and p38 was examined to elucidate the effects of koumine on MAPK pathway. The results showed that the phosphorylation of ERK and p38 MAPK induced by LPS were decreased by koumine, whereas the phosphorylation of JNK induced by LPS was not altered (Figure 3).

### 2.4. Koumine Hampered IκBα and p65 Phosphorylation in LPS-Stimulated RAW264.7 Macrophages

**Figure 4:**
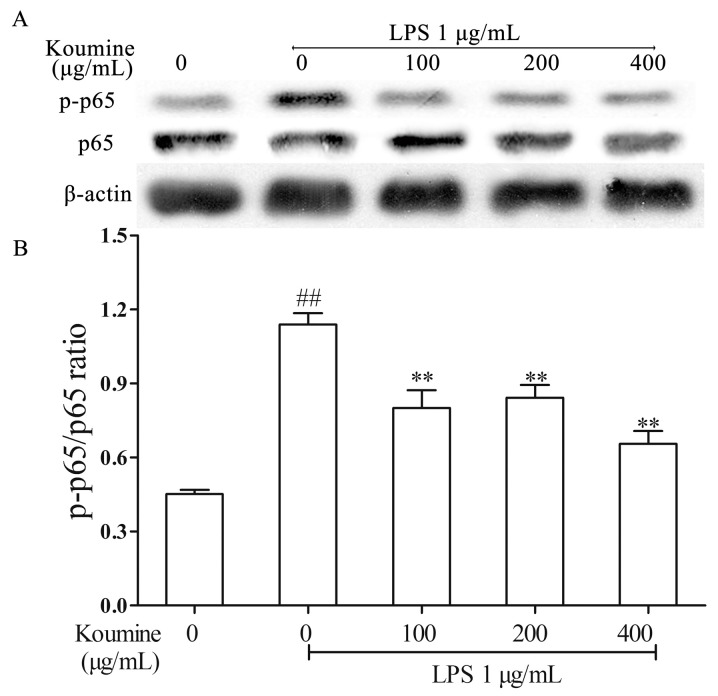
Effect of koumine on the expressions of p-p65 and p65 proteins in LPS stimulated RAW264.7 cells. Cells were pretreated with 100, 200 or 400 μg/mL koumine for 6 h. Subsequently, cells were treated with 1 μg/mL LPS in the mediums containing koumine for 1 h. Equal amounts of p65 and p-p65 proteins in cell lysates were analyzed by Western blot. (**A**) The expression levels of p-p65 and p65; and (**B**) The quantification histogram of p-p65 protein expression normalized by p65. The data were expressed as mean ± standard deviations (SD) (*n* = 3). ^##^ *p <* 0.01 *vs.* control group; ** *p <* 0.01 *vs.* LPS group.

**Figure 5:**
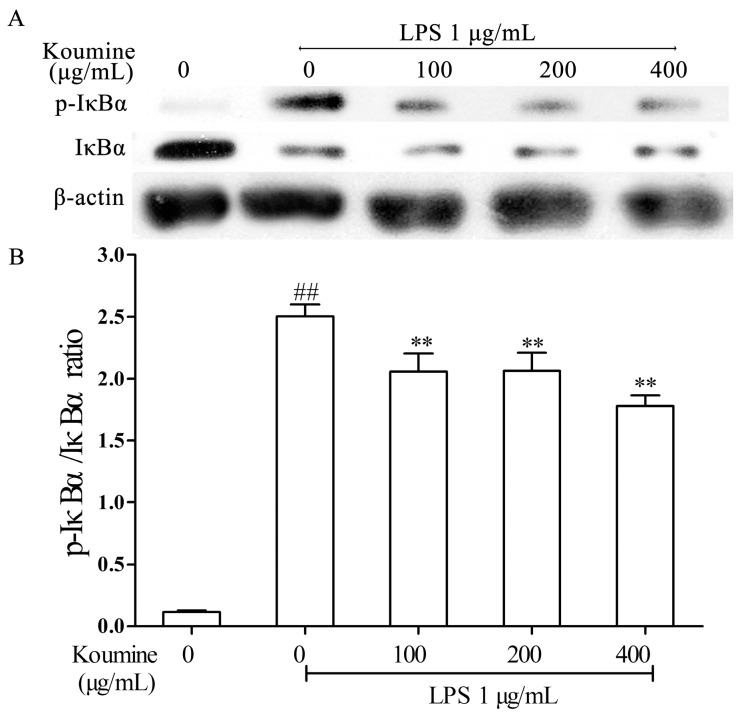
Effect of koumine on the expression of p-IκBα and IκBα in LPS stimulated-RAW264.7 cells. Cells were pretreated with 100, 200 or 400 μg/mL koumine for 6 h. Subsequently, cells were treated with 1 μg/mL LPS in the mediums containing koumine for 30 min. Equal amounts of protein in cell lysates were analyzed by Western blot. The β-actin protein levels were used to confirm that equal amounts of protein were subjected to electrophoresis. (**A**) The expression levels of p-IκBα and IκBα; and (**B**) The quantification histogram of p-IκBα protein expression normalized by β-actin. The data were expressed as mean ± standard deviations (SD) (*n* = 3). ^##^ *p <* 0.01 *vs.* control group; ** *p <* 0.01 *vs.* LPS group.

The effects of koumine in degradation and phosphorylation of IκBα and p65 were determined by measuring IκBα, p-IκBα, p65 and p-p65 proteins expression levels in RAW264.7 macrophages. As shown in the Figure 4 and Figure 5, the IκBα and p65 phosphorylation were remarkably increased by LPS treatment compared with koumine treatment, while the phosphorylation of IκBα and p65 were both stimulated in LPS-treated RAW264.7 cells by various concentrations of koumine. Administration of increasing doses of koumine+LPS are sufficient to diminish the total IκBα levels, although p-IκBα levels were not affected.

### 2.5. Effect of Koumine on DNA-Binding Activity of NF-κB in LPS-Treated RAW264.7 Cells

**Figure 6:**
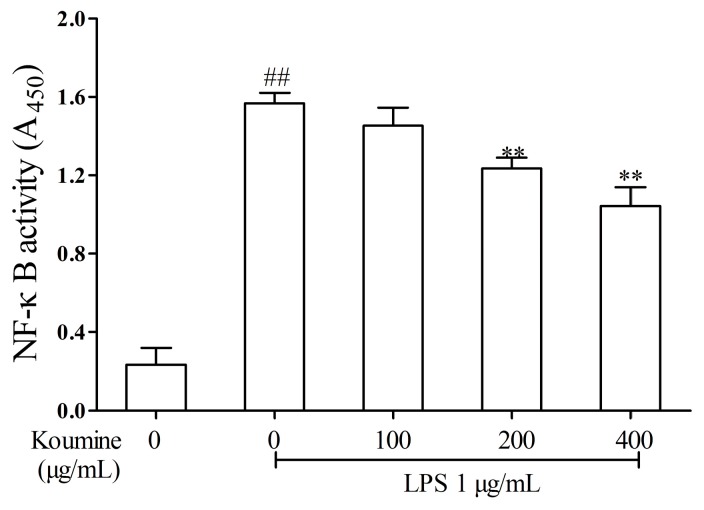
Effect of koumine on NF-κB activity in LPS-stimulated RAW264.7 cells. Cells were pretreated with 100, 200 or 400 μg/mL koumine for 6 h. Subsequently, cells were treated with 1 μg/mL LPS in the mediums containing koumine for 1 h. The nuclear protein extracts were used to measure DNA-binding activity of NF-κB by the Trans AM^TM^ kit for p65. The data were expressed as mean ± standard deviations (SD) (*n* = 3). ^##^ *p <* 0.01 *vs.* control group; ** *p <* 0.01 *vs.* LPS group.

ELISA assay was used to determine koumine’s effect on DNA-binding activity of NF-κB in LPS-treated RAW264.7 macrophages. Compared with the control, the DNA-binding activity of NF-κB following LPS stimulation for 1 h was shown to be significantly higher (*p <* 0.01). On the other hand, LPS-induced NF-κB DNA-binding activity was inhibited by 100, 200 and 400 μg/mL koumine in RAW264.7 cells, as shown in Figure 6.

## 3. Discussion

Compared with the other alkaloids found in *G. elegans* Benth., koumine is the most abundant and is relatively less toxic [21]. Thus, research on its pharmaceutical/medicinal importance has been increasing. For example, its anti-inflammatory activity, analgesic and anti-cancer effects have been reported in some studies [20,22]. Nonetheless, koumine’s anti-inflammatory property and mechanism in RAW264.7 macrophages is not yet well documented. The results of this study reveal that koumine exhibits anti-inflammatory effects in RAW264.7 macrophages stimulated with LPS. These can be explained and attributed to the downregulation of ERK, p38 and NF-κB signal pathways.

The immune system’s initial response to stimuli such as irritants and infection is inflammation. Significant amount of pro-inflammatory mediators are release during the inflammatory process such as NO produced by iNOS, which plays an important role in modulating inflammation and immune cells (e.g., macrophages) [23]. Thus, agents that can inhibit pro-inflammatory mediators have been considered as possible anti-inflammatory agents. Pro-inflammatory cytokines (TNF-α, IL-1β, and IL-6) have been demonstrated to induce expression of iNOS, which lead to tissue damage and multiple organ dysfunctions [24]. It has been suggested that IL-1β and IL-6 appeared to be expressed later than *TNF-α* mRNA with and without LPS stimulation and their levels diminished more slowly [25]. Moreover, TNF-α-induced IL-6 is a prerequisite for increased NO production [26]. The results of the present study showed that koumine noticeably hampered the release of TNF-α, IL-1β and IL-6 in RAW264.7 macrophages stimulated with LPS. These indicate that koumine’s ability to inhibit NO production induced by LPS is connected with iNOS inhibition through decrease production of pro-inflammatory cytokines.

NF-κB also plays an essential part in regulating inflammation and immune responses triggered by extracellular stimuli. The NF-κB family includes c-Rel, p50, p52, p65 (RelA), and RelB, which can form either homodimer or heterodimer with each other. The heterodimer form consisting of a p65 subunit and a p50 or p52 subunit is the most dominant form of NF-κB and contains the transactivation domains that can regulate genes (e.g., iNOS, IL-6, TNF-α, and IL-1β) relevant to immunity and inflammation [27]. IκBa is one of the most important IκB proteins related with transient activation of NF-κB. To further identify the mechanism of koumine in inhibiting IL-6, IL-1β and TNF-α production, this study determined koumine’s effect on activating NF-κB and its influence in phosphorylating IκBα and p65 protein in LPS-induced RAW264.7 macrophages. The results showed that LPS stimulation dramatically increased NF-κB activation, and the phosphorylation of IκBα and p65, while LPS-induced NF-κB activation as well as IκBα and p65 phosphorylation were significantly decreased by pretreatment with koumine. These results imply that koumine’s effects on inflammatory mediator and cytokine productions are regulated, at least in part, by suppressing the NF-κB signaling pathway in LPS-induced RAW264.7 macrophages.

MAPK is a group of serine/threonine protein kinases composed of three subfamilies, namely ERKs, JNKs, and p38 MAPKs. Previously, it has been associated with signaling pathways related to inflammation induced by LPS. P38 can be activated through LPS stimulation and has been speculated to be involved in regulating *iNOS* and *TNF-α* gene expression [28]. JNK was also reported to regulate iNOS expression induced by LPS [29]. In addition, activation of ERK was reported to be associated with LPS-induced macrophage responses, such as increased TNF-α production [30]. In this study, the effects of koumine on MAPKs phosphorylation in LPS-induced RAW264.7 were investigated. The results showed that LPS activated all three types of MAPKs in RAW264.7 cells, which are similar to the results of a previous study [31]. However, it was observed that koumine did not hamper LPS-induced JNK phosphorylation. On the other hand, koumine was observed to hamper ERK phosphorylation, which suggests that inhibition of ERK is due to koumine-induced decrease in TNF-α transcription [30]. In addition, koumine significantly suppressed p38 phosphorylation induced by LPS, which suggests that it is a major mechanism involved in the reduction of LPS-induced iNOS expression and not JNK phosphorylation. These findings demonstrated that phosphorylation of p38 and ERK are involved in koumine’s anti-inflammatory effects on LPS-induced inflammatory cytokine expressions, namely TNF-α, IL-1 and IL-6, and eventual attenuation of subsequent inflammatory response.

## 4. Experimental Section

### 4.1. Materials

Antibodies against iNOS, p-IκBα, IκBα and β-actin were obtained from BD Pharmingen (San Diego, CA, USA). Antibodies against NF-κB p65, p-JNK, p-ERK, p-p38 and p-NF-κB p65 were from Cell Signaling Technology (Danvers, MA, USA). Trans AM™ kit for p65 and nuclear extract kit were purchased from Active Motif (Carlsbad, CA, USA). Amersham Electrogenerated Chemiluminescence (ECL) Advance Western blot detection kit and polyvinylidene fluoride (PVDF) membrane were from GE Heathcare Life (Logan, UT, USA).

### 4.2. Cell Culture

RAW264.7 cells were obtained from Conservation Genetics CAS Kunming Cell Bank, and were grown in Dulbecco’s modified Eagle’s medium (DMEM; Gibco, GrandIsland, NY, USA) supplemented with 10% (*v*/*v*) fetal bovine serum (Gibco, GrandIsland, NY, USA), 100 μg/mL streptomycin (HyClone, Logan, UT, USA) and 100 U/mL penicillin (HyClone) at 37 °C under a humidified 5% CO_2_ incubator.

### 4.3. Assessment of Cell Cytotoxicity

Cell cytotoxicity was determined by MTT (3-(4,5-dimethylthiazol-2-yl)-2,5-diphenyltetrazolium bromide) assay following the method described by Robb *et al.* [32]. To test the cytotoxicity of koumine, cells were incubated with 100, 200 or 400 μg/mL koumine for 24 h. Cells were incubated in 0.5 mg/mL MTT for 4 h in 5%CO_2_. Subsequently, the formazan crystals were dissolved in dimethyl sulfoxide (DMSO), and then plates were read with a microplate reader at 570 nm (MK3, Thermo, Waltham, MA, USA). Cell survival was expressed as the growth of live cells compared with untreated cells. For a positive control, triton X-100 (0.05%) was used as a positive control. Samples were measured in quintuplicate, and this experiment was repeated three separate times.

### 4.4. Measurement of Nitric Oxide and Pro-Inflammatory Cytokines 

Cells were pre-incubated with 100, 200 or 400 μg/mL koumine for 1 h followed by 1 μg/mL LPS treatment. After 18 h, the culture supernatants were collected for NO detection. Briefly, 100 μL cell-free culture media was reacted with 100 μL Griess reagent. The absorbance was obtained at 540 nm. The levels of TNF-α, IL-1β, IL-6, and iNOS were measured by ELISA kits (BD Biosciences, Mississauga, ON, Canada). Samples were measured in triplicate, and this experiment was repeated three separate times.

### 4.5. Western Blot Analysis

Cells were pre-incubated with koumine (100, 200 or 400 μg/mL) for 1 h followed by another 18 h LPS (1 μg/mL) treatment for assaying p-p38, iNOS, p-ERK and phorsphorylated c-Jun N-terminal protein kinase (p-JNK) proteins or pre-cultured with 100, 200 or 400 μg/mL koumine for 6 h, and added with 1 μg/mL LPS in the medium containing koumine for 30 min (for IκBα and p-IκBα), or for 1 h (for p-p65) in the medium containing koumine. The cellular proteins were extracted using lysis buffer (50 mmol/L Tris-HCl, pH 8.0, 150 mmol/L NaCl, 0.5% sodium deoxycholate, 0.1% SDS, 1% NP-40), EDTA-free protease inhibitor cocktail (Roche, Basel, Switzerland) and phosphatase inhibitor cocktail (Roche, Basel, Switzerland). The protein concentrations were determined by the bicinchoninic acid (BCA) assay (Pierce, Rockford, IL, USA) following the manufacturer’s protocol. Equal amounts of protein samples from each cell lysate were separated by SDS-PAGE electrophoresis in a 10% sodium dodecyl sulfate polyacrylamide gel electrophoresis followed by transferred to PVDF membranes. After blocking with 5% skim milk (Yili, Tianjin, China) in PBS for 1 h at room temperature, the membranes were incubated with optimal dilution of the primary antibodies in PBS buffer containing 1% BSA at 4 °C overnight. After washing with PBST, the membrane was incubated with optimal dilution of the secondary antibody for 1 h at room temperature. The protein bands were detected using ECL reagents followed by detected using the ChemiDoc XRS imaging system (Bio-Rad, Hercules, CA, USA). Samples were measured in triplicate.

### 4.6. Nuclear Protein Extracts

Cells were pretreated with 100, 200 or 400 μg/mL koumine for 6 h, and then LPS of 1 μg/mL was added into a koumine-containing medium. After incubation for 30 min or 1 h, cells were gently resuspended in 500 μL hypotonic buffer, then incubated on ice for 15 min. 25 μL of detergent was added followed by centrifugation at 14,000× *g* for 30 s at 4 °C. The pellet was used for nuclear fraction collection. Nuclear pellet was resuspended in 50 μL complete lysis buffer followed by incubation on ice for 30 min. After centrifugation for 10 min, the supernatant was collected and frozen at −80 °C. Samples were measured in triplicate, and this experiment was repeated three separate times.

### 4.7. NF-κB DNA-Binding Assay

Cells (1 × 10^6^ cell/well in 6-well culture plates) were treated by an ELISA kit. Nuclear protein extraction was performed according to the introduction of the kits. Then nuclear protein was treated according to instructions of Trans AM^TM^ NF-κB kit (Active Motif). The optical density was determined at 450 nm by an ELISA reader (MK3, Thermo). Samples were measured in triplicate, and this experiment was repeated three separate times.

### 4.8. Statistical Analysis

Data were expressed as mean ± SD. Data were analyzed using LSD tests and one-way ANOVA using SPSS 19 software (Version 13.0). A *p* value < 0.05 were considered as statistically significant.

## 5. Conclusions

In this study, koumine showed anti-inflammatory activity that is dependent on its capacity to regulate NO, IL-1β, IL-6, and TNF-α production through reduced activation of NF-κB as well as p38 and ERK MAPKs phosphorylation in LPS-stimulated RAW264.7 macrophages. With these mechanisms of action, koumine, therefore, is a potential compound and worthy candidate as treatment for inflammatory diseases.
